# A modified protocol for successful miRNA profiling in human precision-cut lung slices (PCLS)

**DOI:** 10.1186/s13104-021-05674-w

**Published:** 2021-07-02

**Authors:** Monika Niehof, Stella Marie Reamon-Buettner, Olga Danov, Tanja Hansen, Katherina Sewald

**Affiliations:** Department of Preclinical Pharmacology and In Vitro Toxicology, Fraunhofer Institute for Toxicology and Experimental Medicine, Biomedical Research in Endstage and Obstructive Lung Disease Hannover (BREATH), Member of the German Center for Lung Research (DZL), Nikolai-Fuchs-Str. 1, Hannover, 30625 Germany

**Keywords:** miRNA extraction, RTqPCR, Microarray, miRNA array, RNA quality, Lung tissue, Lung material, Ex vivo, PCLS, Organotypic tissue

## Abstract

**Objective:**

Human precision cut lung slices (PCLS) are widely used as an ex vivo model system for drug discovery and development of new therapies. PCLS reflect the functional heterogeneity of lung tissue and possess relevant lung cell types. We thus determined the use of PCLS in studying non-coding RNAs notably miRNAs, which are important gene regulatory molecules. Since miRNAs play key role as mediators of respiratory diseases, they can serve as valuable prognostic or diagnostic biomarkers, and in therapeutic interventions, of lung diseases. A technical limitation though is the vast amount of agarose in PCLS which impedes (mi)RNA extraction by standard procedures. Here we modified our recently published protocol for RNA isolation from PCLS to enable miRNA readouts.

**Results:**

The modified method relies on the separation of lysis and precipitation steps, and a clean-up procedure with specific magnetic beads. We obtained successfully quality miRNA amenable for downstream applications such as RTqPCR and whole transcriptome miRNA analysis. Comparison of miRNA profiles in PCLS with published data from human lung, identified all important miRNAs regulated in IPF, COPD, asthma or lung cancer. Therefore, this shows suitability of the method for analyzing miRNA targets and biomarkers in the valuable human PCLS model.

**Supplementary Information:**

The online version contains supplementary material available at 10.1186/s13104-021-05674-w.

## Introduction

MicroRNAs (miRNAs) are key transcriptional regulators of mRNA in eukaryotic cells. They are among the most abundant classes of gene regulatory molecules and control a wide range of biological functions including cellular proliferation, differentiation and apoptosis [1–3]. miRNAs are non-coding, single-stranded RNAs of 20–23 nucleotides and negatively regulate their mRNA targets by degrading and/or inhibiting protein translation. miRNAs may have a broad function in fine-tuning the protein-coding genes and their discovery has revolutionized our understanding of gene regulation [4–6]. Many studies now link dysregulated miRNA expression to diseases, leading to their increasing importance as biomarkers and therapeutic agents [7–9]. The leading areas of miRNA diagnostics include the cancer field, neurological and cardiovascular diseases [3, 10, 11]. For therapeutic invention, approaches are made using miRNA mimics to inhibit tumor growth [12–15]. Furthermore, chemically-modified antisense oligonucleotides are used to interfere with miRNA function (antagomirs) [16, 17] and their therapeutic efficacy was already shown for example in a cardiovascular disease setting [18–20]. Some RNA therapeutics have been approved or in phase III trials [21]. Accumulating number of investigations show a pivotal role of miRNAs in the pathogenesis of respiratory diseases such as fibrosis, asthma or chronic obstructive pulmonary disease (COPD) [22–27]. Given these central role of miRNAs in lung diseases, they are candidates for prognostic or therapeutic biomarkers and novel therapeutic approaches.

Precision-cut lung slices (PCLS) are widely used for discovery and development of new drugs and therapies in lung diseases [28]. They are thin viable sections of lung tissue which can be cultured under normal cell culture conditions and exposed to agents such as drugs, chemicals, nanoparticles, virus, bacteria or fungi [29]. PCLS reflect the functional heterogeneity of lung tissue and possess relevant cell types including airway and alveolar epithelial cells, smooth muscle cells, endothelial cells, and much more. Active populations of both innate and adaptive immune cells such as alveolar macrophages and T-cells can also be found in varying numbers within precision-cut lung slices. They have been proven to provide highly translational data [30, 31]. During work with PCLS the large amount of agarose in PCLS impedes RNA extraction according to standard procedures. Therefore, we recently published an optimized protocol for RNA isolation from PCLS to perform gene expression analysis in this valuable model [32]. Despite increasing importance of miRNAs, we did not find so far studies describing the isolation and analysis of miRNA derived from PCLS. We thus aimed to establish an optimized protocol for isolating total RNA including miRNA from PCLS. Here we show miRNA from PCLS amenable for downstream applications such as RTqPCR or whole transcriptome miRNA analysis. We also demonstrate the suitability of human PCLS as a model for lung specific miRNA investigations by comparing miRNA profiles of PCLS samples with published data from human lungs.

## Main text

### Methods

#### Human lung tissue and PCLS preparation

Human lung lobes were obtained from male and female patients who underwent lung resection for cancer. Tumor- free tissue was processed immediately on the day of resection. The average age of patients was 70 ± 11 years. PCLS preparation, cultivation and storage are described in detail in Additional file 1.

#### Isolation of total RNA including miRNA

RNA including miRNA was isolated using the Mag MAX mirVana Total RNA Isolation Kit (ThermoFisher Scientific) with several modifications. The detailed protocol and the RNA quality assessment are described in Additional file 1.

#### Quantitative real time RT-PCR analysis (RTqPCR)

RTqPCR for miR-15a was performed using the miScript PCR system (Qiagen) and qPCR reactions were performed using an ABI PRISM 7500 real-time PCR detection system (Applied Biosystems). Detailed conditions and efficiency calculation are described in Additional file 1.

#### Whole transcriptome miRNA analysis and data analysis

Whole transcriptome miRNA analysis was performed using Affymetrix GeneChip® miRNA 4.0 arrays (ThermoFisher Scientific) according to the manufacturer’s instructions. Briefly, 100 ng of total RNA was labeled with biotin using the flashtag™ HSR RNA labeling kit (ThermoFisher Scientific) and then hybridized for 18 h at 48 °C with the array, which was subsequently washed, stained, and read out with a GeneChip® Scanner 3000 7G. The raw data are available at the Gene Expression Omnibus (GEO) site (http://www.ncbi.nlm.nih.gov/geo, accession number for this dataset GSE167705). Quality control of microarray analysis and visualization of the miRNA data were undertaken using metrics and methods contained in Transcriptome Analysis Console Software (TAC 4.0, Thermo Fisher Scientific) as described in detail in Additional file 1.

#### Data comparison with published data from human lung

We searched NCBI PubMed and the Gene Expression Omnibus (GEO) repository for miRNA profiles from human lungs, and, for instance, we downloaded Raw.CEL files from GSE81293 (Expression of miRNA from lung tissue from Systemic Sclerosis patients with interstitial lung disease (SSc-ILD) and healthy controls [33]. Detailed re-analysis and comparison of data was performed as described in Additional file 1. Furthermore, we compared our miRNA profiles with miRNAs that are implicated in respiratory diseases obtained from literature search.

### Results and discussion

#### Application of an optimized protocol for RNA isolation including miRNA from PCLS and assessment of miRNA by RTqPCR

**Fig. 1:**
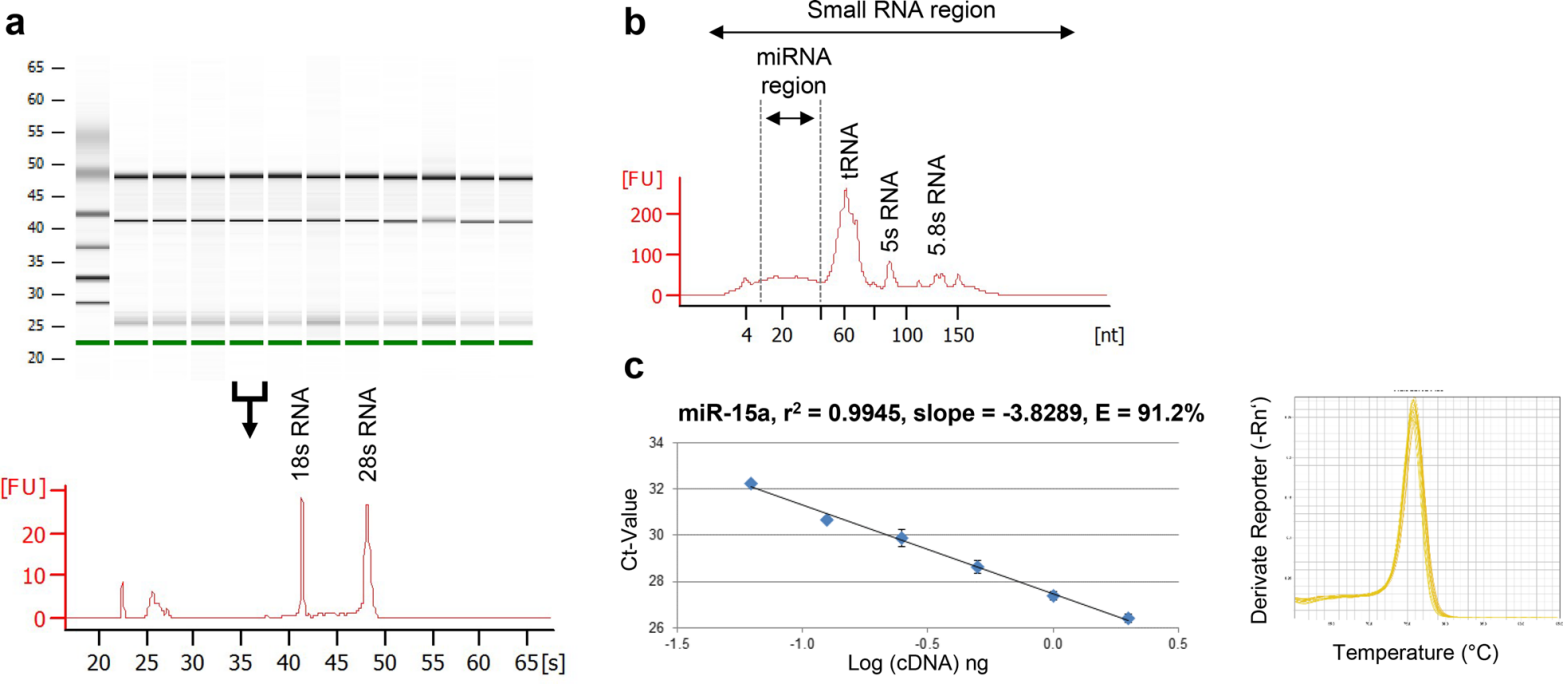
Assessment of RNA quality for human PCLS. **a** Representative bioanalyzer results using Agilent RNA 600 Nano assay showing the integrity of RNA isolated from 10 different human PCLS samples (virtual RNA gel format and electropherogram depicting fluorescence units versus run time in seconds). **b** Representative bioanalyzer results using Agilent Small RNA assay visualizing the presence of small RNAs (electropherogram depicting fluorescence units versus run time in seconds). **c** miScript PCR assay for miR-15a, calibration curve and melting curve using several human PCLS cDNA dilutions

Inspired by our recently published protocol for RNA isolation [32], we established a protocol for isolation of total RNA including miRNA from PCLS which should be suited also for mRNA and miRNA analysis. We used in total 48 PCLS samples from three human donors and carried out treatments with five different substances at three different dose levels as indicated in Additional file 2: Table S1. All samples yielded good RNA quality, i.e. high RNA purity with an A_260_/A_280_ ratio around 2.0 and high RNA integrity with a RIN value around 9.0 (Additional file 2: Table S1). Representative bioanalyzer results for RNA integrity with clear bands at 18 s and 28 s rRNA for n = 10 human PCLS are shown in Fig. 1a. Furthermore, we used Agilent Small RNA assays to visualize the presence of small RNAs in the total RNA preparation (Fig. 1b). As expected for inclusion of small RNAs in samples the miRNA region around 20 nucleotides was visible as a small hill, while great amounts of tRNAs (73–95 nt), small 5 s rRNAs (120 nt) and 5.8 s rRNA (160 nt) could be detected by peaks. Overall, the electropherogram clearly visualized the presence of small RNAs in the samples.

Next we analyzed the expression of miR-15a, which is highly expressed in human tissues, rendering it as an ideal positive control for validating miRNA isolation and RTqPCR quality assessment [6]. RTqPCR analysis of several human PCLS cDNA dilutions resulted in an accurate and linear detection (r^2^ = 0.9945) of miR-15a with high amplification efficiency (E = 91.2%) and a single peak in the melting curve (Fig. 1c). This shows also the suitability of the isolated miRNA derived from human PCLS for RTqPCR based miRNA expression analysis.

#### Genome-wide miRNA profiling in human PCLS and detection of characteristic pulmonary miRNAs

**Fig. 2:**
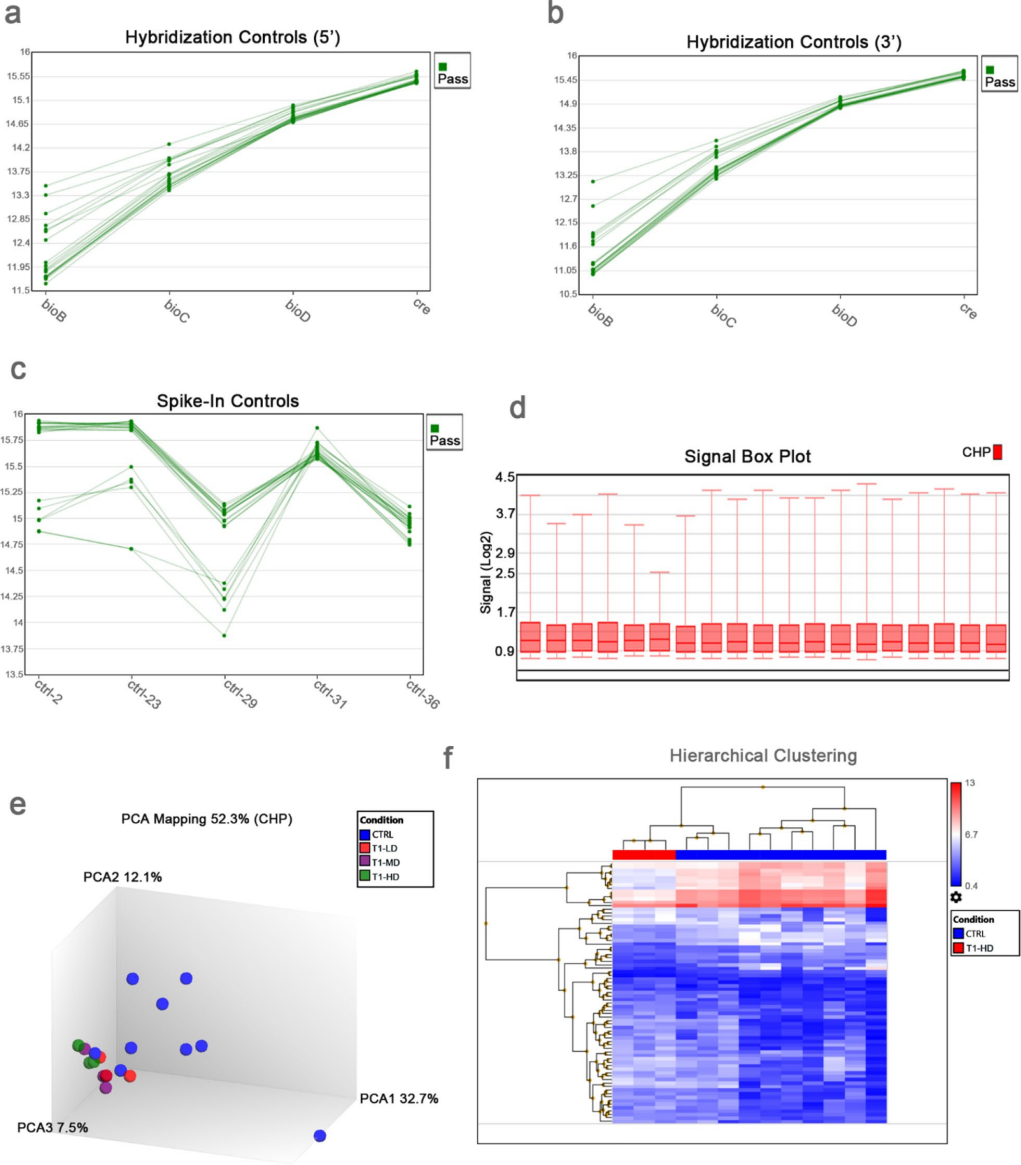
Quality control of miRNA data**.** Affymetrix microarrays were subjected to quality control criteria specified in Transcriptome Analysis Console (TAC 4.0), i.e. (**a**, **b**) 5’ and 3’ hybridization controls, (**c**) spike-in controls and **d** signal box plot. **e** Principal component analysis (PCA) mapping of the miRNA data, showing majority of untreated control samples in one group and treated samples in another group. **f** Hierarchical clustering of filtered genes (Fold Change <  −2 or > 2, P-Value < 0.05, 75 miRNAs, listed in Additional file 5: Table S4) in samples treated with T1-HD vs. untreated control. T1 = Methyl acrylate (CAS 96–33-3), LD = 0.1 mM, MD = 0.3 mM, HD = 1 mM

We performed genome-wide miRNA profiling for untreated control PCLS samples from n = 10 different donors. For comparison purposes with the control samples, we also included for analysis n = 9 PCLS samples, which were treated with three different dose of a chemical compound for risk assessment screening. We analyzed the miRNA data using quality control metrics and visualization methods for gene expression arrays contained in the Transcriptome Analysis Console (TAC 4.0) software. In all the miRNA arrays, the signal values obtained for the 5’ and 3’ hybridization controls, i.e. cRNA of biotin genes bioB, bioC, and bioD from *E. coli* and cre gene from P1 bacteriophage, increased as expected from bioB to cre (Fig. 2a, b). This increase in the signal values for the hybridization controls was a reflection of their increasing relative concentrations. Thus, all the miRNA arrays passed the hybridization control metrics, which were used in monitoring hybridization efficiency. Moreover, all the miRNA arrays also passed the thresholds for spike-in controls consisting of five oligonucleotide probe sets to confirm poly(A) tailing, ligation, and lack of RNAses in the RNA samples (Fig. 2c). A log 2 signal values > 9.96 were obtained for the five oligonucleotide probe sets in all the miRNA arrays. We also inspected the signal box plot of CHP files after normalization using the RMA method and we found that the miRNA arrays did not differ dramatically from each other (Fig. 2d). Figure 2e shows the graph after Principal Component Analysis (PCA) on CHP files, while Fig. 2f shows the hierarchical clustering graph of filtered 75 miRNAs from the comparison of one treatment group vs untreated control. In both PCA and hierarchical clustering graphs, we observed essentially separate clustering of treated samples especially in the high dose (HD) from the untreated ones. Heterogeneity was evident though in the control group presumably due to differences in donor origin.

**Fig. 3:**
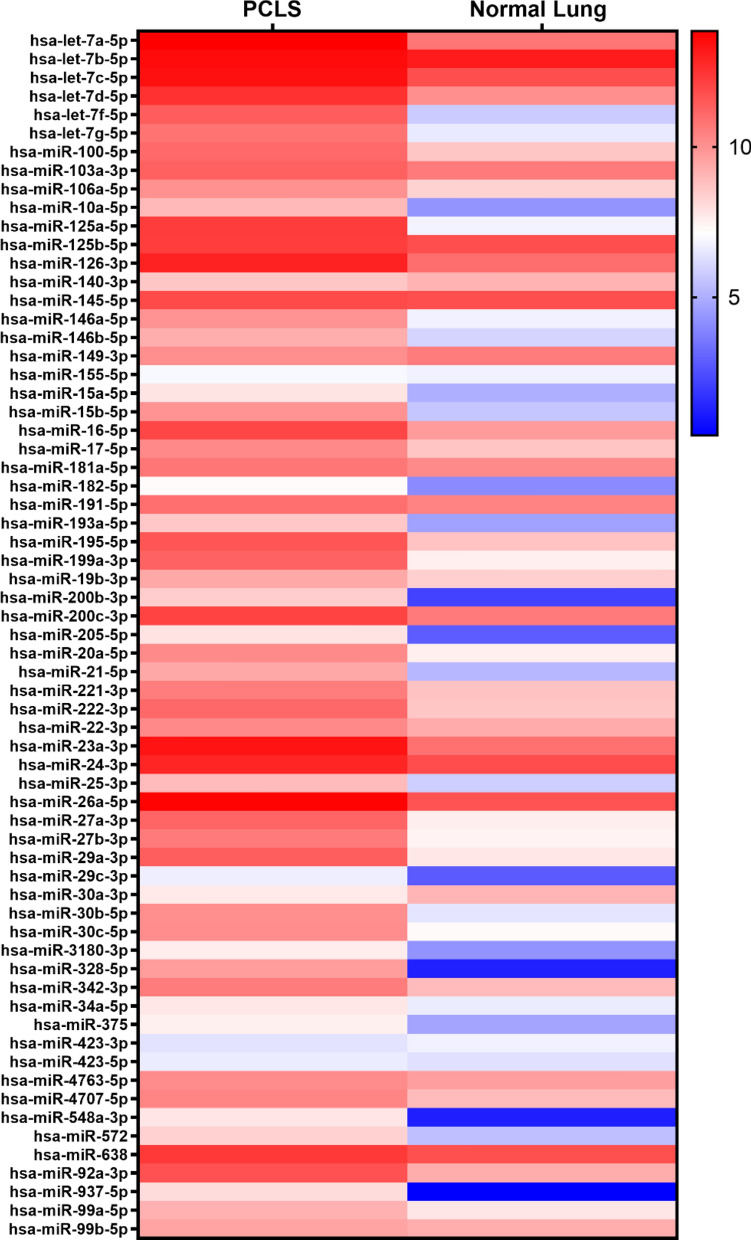
Examples of lung-disease related miRNAs obtained in human PCLS and their level of expression as compared to normal lung samples (GSE81293). Heatmap was based on average signal intensities (log2 values) after microarray analysis and generated using GraphPad Prism 8.3.1

The lung has a unique miRNA expression profile and characteristic miRNA signatures are linked to specific pathological condition [5, 24, 34]. Therefore, we assessed the miRNA profile obtained in human PCLS (Additional file 3: Table S2), according to the maintenance of characteristic pulmonary miRNAs. In detail, our data indicated that miR-195 and miR-200c which are uniquely expressed in lung were detected and that several important miRNAs for physiological lung functions [5, 34, 35] such as let-7, miR-145/146a/b, miR-155, miR-15/16/17, miR-26a and miR-29 were also expressed in human PCLS (Additional file 4: Table S3). We also checked the data for expression of characteristic miRNAs associated with specific pathological condition. Major miRNAs described in idiopathic pulmonary fibrosis with putative functions in aberrant inflammatory responses and in regulation of extra cellular matrix synthesis, collagen expression, matrix metalloproteases (MMP) expression and epithelial-mesenchymal transition [25, 27, 36, 37] such as let-7d, miR-145, miR-199, miR-200b/c, miR-21, miR-26a, miR-29a/c and miR-92a (and several others, see Additional file 4: Table S3) could be detected in human PCLS. miRNAs such as miR-125a/b, miR-145/146a, miR-149, miR-15b and miR-199a (Additional file 4: Table S3) are described as mediators of the TGFß signaling cascade and of genes with impact on the development and progression of COPD, inflammatory response, and airway epithelial repair after injury. They are involved in regulation of airway smooth muscle function, Th2 response activation, macrophage differentiation, recruitment of eosinophils, regulation of matrix metalloproteases, and intensification of unfolded protein responses which contributes to lung cell apoptosis and lung inflammation [25, 35, 38]. miRNAs described with important function in asthma are mainly involved in the promotion of chronic inflammation with effects on T helper 2 (Th2) cells [5, 25] such as miR-106a, miR-126, miR-145/146a, miR-181a, miR-21 and miR-221/222, which were also detectable in PCLS (Additional file 4: Table S3). Furthermore, miRNAs have also been implicated in the regulation of cellular pathways including differentiation, proliferation and survival linked to cancer [5, 23, 35]. Some miRNAs which act as tumor suppressors are downregulated in lung cancer as e.g. members of the let-7 family, miR-100, miR-16 and miR200b, others as e.g. miR-146b, miR-155, miR-21 and miR-221/222 act in promoting tumor formation and were also detected (Additional file 4: Table S3). Overall, we show good comparison of our miRNA dataset (Additional file 3: Table S2) with published data from healthy human lung (GSE81293) [33] (Fig. 3). Indeed, of the lung disease-associated miRNAs compared, 59 of 65 miRNAs exhibited more or less similar expression in PCLS and in the lung tissue.

### Conclusion

In summary, the presented protocol resulted in miRNA of high quality for transcriptome wide analysis and detection of important miRNAs for several pulmonary pathophysiological condition. Therefore, this method will be well suited to analyze miRNA targets and biomarkers in the human PCLS model.

## Limitations

It would be desirable to compare our PCLS datasets with more datasets of healthy human lungs from the literature to confirm our results. However, as of today in the GEO data base only older Affymetrix array versions are additionally available and the TAC 4.0 software does not allow matching of these miRNA probe IDs with the current Affymetrix GeneChip® miRNA arrays version 4.0. Furthermore, the number of donors for PCLS datasets was limited. However, we feel confident that the presented protocol is suitable for downstream applications because it overcomes the limitations of miRNA isolation from PCLS based on agarose and that it resulted in the detection of the most characteristic pulmonary miRNAs.

## Supplementary Information

**Additional file 1**: Methods.

**Additional file 2: Table S1.** RNA yield from huPCLS after different treatments.

**Additional file 3: Table S2.** List of filtered miRNAs obtained in human precision lung cut slices (PCLS).

**Additional file 4: Table S3.** A selected list of some miRNAs detected in human PCLS with already described function in pulmonary diseases.

**Additional file 5: Table S4.** List of filtered 75 differentially-expressed miRNAs in comparison T1-HD versus control used as basis during hierarchical clustering analysis.

## Data Availability

The datasets generated and analyzed during the current study are available at GEO database GSE167705 and GSE81293 [33].

## Abbreviations

COPD: Chronic obstructive pulmonary disease
Cq: Quantification cycle
DMEM: Dulbecco’s Modified Eagle Medium
EBSS: Earle’s Balanced Salt Solution
HD: High dose
LD: Low dose
MD: Mid dose
MMP: Matrix metalloproteases
nt: Nucleotides
PCA: Principal component analysis
PCLS: Precision-cut lung slices
RIN: RNA integrity number
qPCR: Quantitative polymerase chain reaction
RT: Reverse transcriptase
T: Treatment
Th2 cells: T helper 2 cells
