# Supplementary material for: A modified protocol for successful miRNA profiling in human precision-cut lung slices (PCLS)
Source: BMC Res Notes. 2021 Jul 2;14:255. doi: 10.1186/s13104-021-05674-w (PMC8252208; doi:10.1186/s13104-021-05674-w)
Supplement: Supplementary file 1 — Additional file 1: Methods. [file 13104_2021_5674_MOESM1_ESM.docx]

**Niehof et al. A modified protocol for successful miRNA profiling in human precision-cut lung slices (PCLS)**

**Additional file 1. Methods.**

**PCLS preparation, cultivation, and storage**

Human lungs were filled ex situ and PCLS were prepared as previously described [1–3]. Briefly, the trachea was cannulated and the lungs were filled up with 37 °C-warm, 2 % low-gelling agarose medium solution (Sigma-Aldrich, Munich, Germany). After polymerization of agarose, tissue cores were cut into 200- to 300-µm-thick slices using a Krumdieck tissue slicer (Alabama Research and Development, Munford, AL, USA) filled with 4 °C-cold EBSS (Sigma-Aldrich, Munich, Germany). Subsequently, precision-cut lung slices were incubated in DMEM (Dulbecco’s modified Eagle’s medium/nutrient mixture F-12 Ham (DMEM, pH 7.2-7.4) with L-glutamine and 15 mM HEPES (4-(2-hydroxyethyl)-1-piperazineethanesulfonic acid) without phenol red and fetal bovine serum supplied from Gibco™ (Life Technologies/Thermo Fisher Scientific, Dreieich, Germany). The culture medium was supplemented with 100 units/mL penicillin and streptomycin (Lonza, Verviers, Belgium). PCLS were cultured for 72 h under standard cell culture conditions (37 °C, 5 % CO2, 100 % humidity). PCLS preparation steps are summarized in Additonal Figure 1. Two PCLS were cultured together in 500 µL DMEM as described previously [2]. Two slices were pooled and immediately transferred into liquid nitrogen and subsequently stored at -80 °C.

**Additional Figure 1. Preparation steps of human PCLS.** a) Lung lobe was inflated by the agarose medium solution and solidified on ice. b) Tissue cores were sliced in PCLS using a Krumdieck tissue slicer. c) Two PCLS per well were placed into a 24 well plate prior to experiment. d) PCLS were cultured under cell culture conditions prior to miRNA isolation.

**Isolation of total RNA including miRNA and quality assessment**

Our protocol is based on carefully separated lysis and a phenol-chlorofrom precipation step followed by a RNA purification procedure using magnetic beads from the Mag MAX mirVana Total RNA Isolation Kit (ThermoFisher Scientific, Dreieich, Germany). In detail, RLT lysis buffer (Qiagen, Hilden, Germany) was added and PCLS were disrupted and homogenized using an Ultra-Turrax® (T10basic, IKA, Staufen, Germany) for 20 s. The homogenate was transferred to 1 volume of phenol/chloroform, carefully shaken for 30 s, and centrifuged for 5 min at 12,000xg. Subsequently, 1 volume of chloroform/isoamyl alcohol was added, again carefully shaken for 30 s, and centrifuged for 5 min at 12,000xg. The aqueous phase was transferred to a new tube and 1 volume of isopropanol was added. RNA binding beads mix (Mag MAX mirVana Total RNA Isolation Kit, ThermoFisher Scientific, Dreieich, Germany) was generated and added to the samples as indicated by the manufacturer. Next, all samples were transferred into a 96 well processing plate contained in the kit. All further clean up steps including a DNase treatment were performed according to the manufacturer’s recommendation. Total RNA was eluted in pre-heated (37°C) elution buffer (from the kit), pooled from two samples (corresponding to four PCLS in total) and stored at -80 °C. RNA concentration (A260) and purity (A260/A280 ratio) were measured by spectrophotometry (NanoDrop™ 2000 Spectrophotometer, software version 1.6.198, ThermoFisher Scientific, Dreieich, Germany). The RNA 600 Nano assay (Agilent Technologies, Ratingen, Germany) was used for integrity assessment of RNA samples and the Small RNA assay (Agilent Technologies, Ratingen, Germany) was used to visualize small RNAs between 6 and 150 nucleotides. Both assays were analyzed on the Agilent 2100 Bioanalyzer® (Agilent Technologies, Ratingen, Germany).

**Quantitative real time RT-PCR analysis (RTqPCR)**

RTqPCR for miR-15a was performed using the miScript PCR system (Qiagen, Hilden, Germany). The miScript system covers all the steps of conversion of the RNA into cDNA to detection of miRNA in SYBR® Green-based real-time PCR. cDNA was prepared in a reverse transcription reaction using miScript HiSpec buffer. RTqPCR was performed using a human miR-15a-specific miScript primer assay (forward primer) and the miScript universal primer (reverse primer) with QuantiTect SYBR® Green PCR master mix. qPCR reactions were performed using an ABI PRISM 7500 real-time PCR detection system (Applied Biosystems) with the following conditions: 15 min 95 °C; 15 s 94 °C/30 s 55 °C/30 s 70 °C, for 40 cycles; and 15 s 95 °C/1 min 60 °C/ 30 s 95 °C for the melting curve. At the end of each extension phase, fluorescence was recorded and at the end of a run quantification cycles (Cq) were determined for each sample. Serial dilutions of RT reactions were prepared in triplicate (from 2 ng to 0.0625 ng) and samples were analyzed by qPCR to measure Cq values. A plot of Cq values versus logarithm of target concentrations resulted in a standard curve, which was used for efficiency calculation (10(-1/slope) – 1, corresponding to 100%) [4–6].

**Data analysis**

Quality control of microarray analysis and visualization of the miRNA data were undertaken using metrics and methods contained in Transcriptome Analysis Console Software (TAC 4.0, Thermo Fisher Scientific, Dreieich, Germany). Quality control metrics include hybridization and RNA spike-in controls, as well as visualization methods of gene expression data such as signal box plots, principal component analysis (PCA), and hierarchical clustering. The hybridization controls are composed of a mixture of biotinylated and fragmented cRNA of bioB, bioC, and bioD from E. coli and cre from P1 bacteriophage in staggered concentrations. The hybridization controls are high-quality controls used for monitoring array hybridization, washing, and staining for reproducible results. The hybridization controls are spiked into the hybridization cocktail, independent of RNA sample preparation, and are thus used to evaluate sample hybridization efficiency on eukaryotic gene expression arrays. The RNA spike-in controls consist of five oligos to confirm poly(A) tailing, ligation, and lack of RNAses in the RNA sample. Oligos 2, 23, and 29 are RNA, and confirm poly(A) tailing and ligation. Oligo 31 is poly(A) RNA, and confirms ligation. Oligo 36 is poly(dA) DNA, and confirms ligation and lack of RNAses in the RNA sample. Normalization and summarization of the microarray data were performed using the Robust Multi-array Average (RMA) method. Furthermore, we also included a treatment group to enable characterization of the miRNA profiles of control donors using methods for gene expression arrays in the TAC software. Differential gene expression was obtained with the default filter criteria Fold Change: > 2 or < -2; and P-val: < 0.05.

**Data comparison with published data from human lung**

To compare the miRNA profiles of control donors with those from published or publicly available datasets from human lungs, we created a list of miRNAs using a cut-off filter of average log 2 signal intensity > 6. We then searched NCBI PubMed and the Gene Expression Omnibus (GEO) repository for miRNA profiles from human lungs, and determined which of the identified miRNAs in the PCLS control samples were also expressed in those human lungs. For instance, for comparison, we downloaded Raw.CEL files from GSE81293 (Expression of miRNA from lung tissue from Systemic Sclerosis patients with interstitial lung disease (SSc-ILD) and healthy controls [7]. We re-analyzed the CEL files using TAC Software and likewise created a list of miRNAs from the healthy controls. On the basis of average log 2 signal intensities obtained in PCLS control samples and healthy controls (GSE81293), a heatmap was generated manually on a list of lung-disease associated miRNAs using GraphPad Prism 8.3.1.

References

1. Henjakovic M, Sewald K, Switalla S, Kaiser D, Müller M, Veres TZ, et al. Ex vivo testing of immune responses in precision-cut lung slices. Toxicol Appl Pharmacol. 2008;231:68–76. doi:10.1016/j.taap.2008.04.003.

2. Switalla S, Lauenstein L, Prenzler F, Knothe S, Förster C, Fieguth H-G, et al. Natural innate cytokine response to immunomodulators and adjuvants in human precision-cut lung slices. Toxicol Appl Pharmacol. 2010;246:107–15. doi:10.1016/j.taap.2010.04.010.

3. Neuhaus V, Danov O, Konzok S, Obernolte H, Dehmel S, Braubach P, et al. Assessment of the Cytotoxic and Immunomodulatory Effects of Substances in Human Precision-cut Lung Slices. J Vis Exp 2018. doi:10.3791/57042.

4. Svec D, Tichopad A, Novosadova V, Pfaffl MW, Kubista M. How good is a PCR efficiency estimate: Recommendations for precise and robust qPCR efficiency assessments. Biomol Detect Quantif. 2015;3:9–16. doi:10.1016/j.bdq.2015.01.005.

5. Bustin SA, Benes V, Garson JA, Hellemans J, Huggett J, Kubista M, et al. The MIQE guidelines: minimum information for publication of quantitative real-time PCR experiments. Clin Chem. 2009;55:611–22. doi:10.1373/clinchem.2008.112797.

6. Pfaffl MW. A new mathematical model for relative quantification in real-time RT-PCR. Nucleic Acids Res. 2001;29:e45. doi:10.1093/nar/29.9.e45.

7. Christmann RB, Wooten A, Sampaio-Barros P, Borges CL, Carvalho CRR, Kairalla RA, et al. miR-155 in the progression of lung fibrosis in systemic sclerosis. Arthritis Res Ther. 2016;18:155. doi:10.1186/s13075-016-1054-6.
