# Supplementary material for: A modified protocol for successful miRNA profiling in human precision-cut lung slices (PCLS)
Source: BMC Res Notes. 2021 Jul 2;14:255. doi: 10.1186/s13104-021-05674-w (PMC8252208; doi:10.1186/s13104-021-05674-w)
Supplement: Supplementary file 2 — Additional file 2: Table S1. RNA yield from huPCLS after different treatments. [file 13104_2021_5674_MOESM2_ESM.docx]

**Niehof et al. A modified protocol for successful miRNA profiling in human precision-cut lung slices (PCLS)**

**Additional file 2. Table S1.**

**Table S1.** RNA yield from human PCLS after different treatments.

|  | **RNA yield**  **(µg)** | | **Absorbance 260/280** | | **RIN** | |
| --- | --- | --- | --- | --- | --- | --- |
| **Samples** | **avg.** | **min.** | **avg.** | **min.** | **avg.** | **min.** |
| Control | 3.15 | 2.87 | 2.06 | 2.04 | 9.5 | 9.0 |
| T1-LD | 2.76 | 2.12 | 2.03 | 1.97 | 9.4 | 9.3 |
| T1-MD | 2.25 | 1.69 | 2.03 | 2.02 | 9.3 | 9.2 |
| T1-HD | 1.61 | 1.29 | 2.02 | 2.01 | 9.3 | 9.0 |
| T2-LD | 1.71 | 1.29 | 2.03 | 1.97 | 8.5 | 6.4 |
| T2-MD | 3.29 | 1.94 | 2.06 | 2.05 | 9.4 | 9.1 |
| T2-HD | 2.35 | 1.64 | 2.00 | 1.97 | 8.4 | 7.5 |
| T3-LD | 2.04 | 0.93 | 2.02 | 1.96 | 9.5 | 9.2 |
| T3-MD | 3.02 | 1.32 | 2.01 | 1.95 | 9.2 | 8.8 |
| T3-HD | 1.87 | 0.66 | 1.99 | 1.94 | 9.3 | 9.1 |
| T4-LD | 2.07 | 1.44 | 2.04 | 2.05 | 9.6 | 9.3 |
| T4-MD | 3.44 | 3.05 | 2.04 | 2.01 | 9.7 | 9.5 |
| T4-HD | 3.27 | 2.69 | 2.02 | 2.00 | 8.9 | 8.2 |
| T5-LD | 2.10 | 0.86 | 1.99 | 1.91 | 8.8 | 8.2 |
| T5-MD | 2.00 | 0.82 | 1.98 | 1.93 | 8.8 | 7.7 |
| T5-HD | 2.92 | 2.87 | 2.02 | 2.04 | 9.4 | 9.0 |
| Mean | 2.49 ± 0.62 | | 2.02 ± 0.02 | | 9.2 ± 0.4 | |

All samples derived from three different donors. The mean is indicated as ± STABW. T1 to T5 indicate treatment with five different substances. T1 = Methyl acrylate (CAS 96-33-3), T2 = Ethyl acrylate (CAS 140-88-5), T3 = n-Propyl acrylate (CAS 925-60-0), T4 = Butyl acrylate (CAS 141-32-2), T5 = tert-Butyl alcohol (CAS 75-65-0). T1 – T4: LD = 0.1 mM, MD = 0.3 mM, HD = 1 mM. T5: LD = 0.1 mM, MD = 1 mM, HD = 10 mM. LD = low dose, MD = mid dose, HD = high dose.
