# Supplementary material for: A modified protocol for successful miRNA profiling in human precision-cut lung slices (PCLS)
Source: BMC Res Notes. 2021 Jul 2;14:255. doi: 10.1186/s13104-021-05674-w (PMC8252208; doi:10.1186/s13104-021-05674-w)
Supplement: Supplementary file 3 — Additional file 3: Table S2. List of filtered miRNAs obtained in human precision lung cut slices (PCLS). [file 13104_2021_5674_MOESM3_ESM.docx]

**Niehof et al. A modified protocol for successful miRNA profiling in human precision-cut lung slices (PCLS)**

**Additional file 3. Table S2.**

**Table S2.** List of filtered miRNAs obtained in human precision lung cut slices (PCLS).

| **Array ID** | **Probe Set Name** | **Transcript ID** | **Average Signal Intensity (log2)** |
| --- | --- | --- | --- |
| 20500112 | MIMAT0000062_st | hsa-let-7a-5p | 13.85 |
| 20500152 | MIMAT0000082_st | hsa-miR-26a-5p | 13.79 |
| 20519048 | MIMAT0019337_st | hsa-miR-3960 | 13.70 |
| 20500115 | MIMAT0000063_st | hsa-let-7b-5p | 13.56 |
| 20500117 | MIMAT0000064_st | hsa-let-7c-5p | 13.41 |
| 20517919 | MIMAT0018087_st | hsa-miR-3665 | 13.41 |
| 20500146 | MIMAT0000078_st | hsa-miR-23a-3p | 13.33 |
| 20523021 | MIMAT0023714_st | hsa-miR-6089 | 13.25 |
| 20523022 | MIMAT0023715_st | hsa-miR-6090 | 13.13 |
| 20519679 | MIMAT0019956_st | hsa-miR-4787-5p | 13.09 |
| 20500721 | MIMAT0000418_st | hsa-miR-23b-3p | 13.02 |
| 20500769 | MIMAT0000445_st | hsa-miR-126-3p | 12.93 |
| 20528733 | MIMAT0030019_st | hsa-miR-7704 | 12.91 |
| 20500148 | MIMAT0000080_st | hsa-miR-24-3p | 12.85 |
| 20523019 | MIMAT0023712_st | hsa-miR-6087 | 12.80 |
| 20518851 | MIMAT0018993_st | hsa-miR-4466 | 12.77 |
| 20524035 | MIMAT0024598_st | hsa-miR-6125 | 12.74 |
| 20525699 | MIMAT0027638_st | hsa-miR-6869-5p | 12.63 |
| 20509237 | MIMAT0007892_st | hsa-miR-1915-3p | 12.58 |
| 20500119 | MIMAT0000065_st | hsa-let-7d-5p | 12.55 |
| 20525420 | MIMAT0027359_st | hsa-miR-6729-5p | 12.47 |
| 20523020 | MIMAT0023713_st | hsa-miR-6088 | 12.39 |
| 20529779 | MIMAT0030996_st | hsa-miR-8069 | 12.38 |
| 20504391 | MIMAT0003308_st | hsa-miR-638 | 12.36 |
| 20500765 | MIMAT0000443_st | hsa-miR-125a-5p | 12.25 |
| 20500730 | MIMAT0000423_st | hsa-miR-125b-5p | 12.24 |
| 20518892 | MIMAT0019032_st | hsa-miR-4497 | 12.20 |
| 20500752 | MIMAT0000435_st | hsa-miR-143-3p | 12.18 |
| 20518882 | MIMAT0019022_st | hsa-miR-4488 | 12.18 |
| 20514163 | MIMAT0013802_st | hsa-miR-2861 | 12.14 |
| 20517907 | MIMAT0018076_st | hsa-miR-3656 | 12.11 |
| 20501036 | MIMAT0000617_st | hsa-miR-200c-3p | 12.07 |
| 20525416 | MIMAT0027355_st | hsa-miR-6727-5p | 12.04 |
| 20515638 | MIMAT0015080_st | hsa-miR-3196 | 12.01 |
| 20500128 | MIMAT0000069_st | hsa-miR-16-5p | 11.99 |
| 20518913 | MIMAT0019053_st | hsa-miR-4516 | 11.98 |
| 20500121 | MIMAT0000066_st | hsa-let-7e-5p | 11.91 |
| 20500755 | MIMAT0000437_st | hsa-miR-145-5p | 11.89 |
| 20506787 | MIMAT0022946_st | hsa-miR-1237-5p | 11.72 |
| 20500171 | MIMAT0000092_st | hsa-miR-92a-3p | 11.71 |
| 20529782 | MIMAT0030999_st | hsa-miR-8072 | 11.69 |
| 20525396 | MIMAT0025856_st | hsa-miR-6724-5p | 11.65 |
| 20522537 | MIMAT0023252_st | hsa-miR-5787 | 11.64 |
| 20500798 | MIMAT0000461_st | hsa-miR-195-5p | 11.59 |
| 20526174 | MIMAT0028113_st | hsa-miR-7108-5p | 11.58 |
| 20509224 | MIMAT0007881_st | hsa-miR-1908-5p | 11.48 |
| 20518905 | MIMAT0019045_st | hsa-miR-4508 | 11.43 |
| 20500123 | MIMAT0000067_st | hsa-let-7f-5p | 11.39 |
| 20525533 | MIMAT0027472_st | hsa-miR-6786-5p | 11.38 |
| 20500161 | MIMAT0010313_st | hsa-miR-29a-3p | 11.35 |
| 20504586 | MIMAT0000086_st | hsa-miR-762 | 11.35 |
| 20500191 | MIMAT0000101_st | hsa-miR-103a-3p | 11.27 |
| 20500400 | MIMAT0000232_st | hsa-miR-199a-3p | 11.26 |
| 20500458 | MIMAT0004563_st | hsa-miR-199b-3p | 11.26 |
| 20525561 | MIMAT0027500_st | hsa-miR-6800-5p | 11.22 |
| 20507742 | MIMAT0007347_st | hsa-miR-1469 | 11.21 |
| 20518629 | MIMAT0019229_st | hsa-miR-3940-5p | 11.19 |
| 20500157 | MIMAT0000084_st | hsa-miR-27a-3p | 11.17 |
| 20500162 | MIMAT0000087_st | hsa-miR-30a-5p | 11.17 |
| 20500196 | MIMAT0000104_st | hsa-miR-107 | 11.13 |
| 20500486 | MIMAT0000279_st | hsa-miR-222-3p | 11.09 |
| 20500183 | MIMAT0000098_st | hsa-miR-100-5p | 11.08 |
| 20500715 | MIMAT0000415_st | hsa-let-7i-5p | 11.04 |
| 20520351 | MIMAT0022742_st | hsa-miR-1273g-3p | 11.03 |
| 20501286 | MIMAT0004697_st | hsa-miR-151a-5p | 10.96 |
| 20520198 | MIMAT0021021_st | hsa-miR-5001-5p | 10.95 |
| 20500781 | MIMAT0004609_st | hsa-miR-149-3p | 10.90 |
| 20500761 | MIMAT0000440_st | hsa-miR-191-5p | 10.90 |
| 20519493 | MIMAT0019775_st | hsa-miR-4687-3p | 10.88 |
| 20525567 | MIMAT0027506_st | hsa-miR-6803-5p | 10.84 |
| 20518878 | MIMAT0019018_st | hsa-miR-4484 | 10.83 |
| 20500713 | MIMAT0000414_st | hsa-let-7g-5p | 10.81 |
| 20519600 | MIMAT0019878_st | hsa-miR-4745-5p | 10.81 |
| 20525543 | MIMAT0027482_st | hsa-miR-6791-5p | 10.78 |
| 20500444 | MIMAT0000256_st | hsa-miR-181a-5p | 10.73 |
| 20525603 | MIMAT0027542_st | hsa-miR-6821-5p | 10.72 |
| 20525661 | MIMAT0027600_st | hsa-miR-6850-5p | 10.72 |
| 20525593 | MIMAT0027532_st | hsa-miR-6816-5p | 10.70 |
| 20500848 | MIMAT0000510_st | hsa-miR-320a | 10.68 |
| 20518852 | MIMAT0018994_st | hsa-miR-4467 | 10.63 |
| 20500723 | MIMAT0005793_st | hsa-miR-27b-3p | 10.61 |
| 20504563 | MIMAT0000419_st | hsa-miR-320c | 10.61 |
| 20501280 | MIMAT0000753_st | hsa-miR-342-3p | 10.56 |
| 20504562 | MIMAT0005792_st | hsa-miR-320b | 10.55 |
| 20500484 | MIMAT0000278_st | hsa-miR-221-3p | 10.53 |
| 20518933 | MIMAT0019071_st | hsa-miR-4532 | 10.44 |
| 20506774 | MIMAT0005582_st | hsa-miR-1228-5p | 10.43 |
| 20524036 | MIMAT0003326_st | hsa-miR-6126 | 10.37 |
| 20504413 | MIMAT0024599_st | hsa-miR-663a | 10.37 |
| 20525539 | MIMAT0027478_st | hsa-miR-6789-5p | 10.37 |
| 20519525 | MIMAT0019807_st | hsa-miR-4707-5p | 10.32 |
| 20500782 | MIMAT0019859_st | hsa-miR-150-5p | 10.30 |
| 20519580 | MIMAT0000451_st | hsa-miR-4734 | 10.30 |
| 20506771 | MIMAT0022941_st | hsa-miR-1227-5p | 10.27 |
| 20525571 | MIMAT0027510_st | hsa-miR-6805-5p | 10.24 |
| 20500144 | MIMAT0000077_st | hsa-miR-22-3p | 10.23 |
| 20500424 | MIMAT0000245_st | hsa-miR-30d-5p | 10.23 |
| 20500130 | MIMAT0000070_st | hsa-miR-17-5p | 10.22 |
| 20517736 | MIMAT0016907_st | hsa-miR-4281 | 10.22 |
| 20500139 | MIMAT0000075_st | hsa-miR-20a-5p | 10.21 |
| 20525426 | MIMAT0027365_st | hsa-miR-6732-5p | 10.21 |
| 20515607 | MIMAT0015055_st | hsa-miR-3178 | 10.18 |
| 20519636 | MIMAT0019913_st | hsa-miR-4763-3p | 10.15 |
| 20500422 | MIMAT0000244_st | hsa-miR-30c-5p | 10.11 |
| 20500724 | MIMAT0000420_st | hsa-miR-30b-5p | 10.06 |
| 20500194 | MIMAT0000103_st | hsa-miR-106a-5p | 10.01 |
| 20523000 | MIMAT0023693_st | hsa-miR-6068 | 10.01 |
| 20500718 | MIMAT0000417_st | hsa-miR-15b-5p | 9.99 |
| 20500778 | MIMAT0000449_st | hsa-miR-146a-5p | 9.96 |
| 20525677 | MIMAT0027616_st | hsa-miR-6858-5p | 9.93 |
| 20509070 | MIMAT0006764_st | hsa-miR-320d | 9.88 |
| 20504187 | MIMAT0004784_st | hsa-miR-455-3p | 9.78 |
| 20501277 | MIMAT0026486_st | hsa-miR-328-5p | 9.73 |
| 20525491 | MIMAT0027430_st | hsa-miR-6765-5p | 9.71 |
| 20525448 | MIMAT0027387_st | hsa-miR-6743-5p | 9.70 |
| 20501197 | MIMAT0000703_st | hsa-miR-361-5p | 9.66 |
| 20518931 | MIMAT0019069_st | hsa-miR-4530 | 9.63 |
| 20517835 | MIMAT0018002_st | hsa-miR-3621 | 9.58 |
| 20517729 | MIMAT0016900_st | hsa-miR-4270 | 9.57 |
| 20525394 | MIMAT0000689_st | hsa-miR-6722-3p | 9.54 |
| 20501176 | MIMAT0025854_st | hsa-miR-99b-5p | 9.54 |
| 20518845 | MIMAT0018987_st | hsa-miR-4463 | 9.53 |
| 20519474 | MIMAT0019756_st | hsa-miR-4674 | 9.48 |
| 20525465 | MIMAT0027404_st | hsa-miR-6752-5p | 9.47 |
| 20515617 | MIMAT0015065_st | hsa-miR-3185 | 9.46 |
| 20500137 | MIMAT0000074_st | hsa-miR-19b-3p | 9.42 |
| 20500141 | MIMAT0000076_st | hsa-miR-21-5p | 9.41 |
| 20519494 | MIMAT0027038_st | hsa-miR-1343-5p | 9.27 |
| 20503793 | MIMAT0002809_st | hsa-miR-146b-5p | 9.25 |
| 20518887 | MIMAT0019027_st | hsa-miR-4492 | 9.25 |
| 20519589 | MIMAT0019868_st | hsa-miR-4739 | 9.23 |
| 20500181 | MIMAT0000097_st | hsa-miR-99a-5p | 9.16 |
| 20518834 | MIMAT0018976_st | hsa-miR-4454 | 9.03 |
| 20500438 | MIMAT0000253_st | hsa-miR-10a-5p | 8.95 |
| 20500151 | MIMAT0019715_st | hsa-miR-25-3p | 8.91 |
| 20519433 | MIMAT0000081_st | hsa-miR-4651 | 8.91 |
| 20525511 | MIMAT0027450_st | hsa-miR-6775-5p | 8.85 |
| 20504561 | MIMAT0019788_st | hsa-miR-151b | 8.84 |
| 20519507 | MIMAT0010214_st | hsa-miR-4695-5p | 8.84 |
| 20515550 | MIMAT0015010_st | hsa-miR-3141 | 8.79 |
| 20506837 | MIMAT0005898_st | hsa-miR-1246 | 8.78 |
| 20501157 | MIMAT0000680_st | hsa-miR-106b-5p | 8.73 |
| 20529785 | MIMAT0031002_st | hsa-miR-8075 | 8.71 |
| 20511558 | MIMAT0011158_st | hsa-miR-2115-5p | 8.66 |
| 20500735 | MIMAT0000425_st | hsa-miR-130a-3p | 8.63 |
| 20504298 | MIMAT0003239_st | hsa-miR-574-3p | 8.62 |
| 20500746 | MIMAT0004597_st | hsa-miR-140-3p | 8.61 |
| 20519592 | MIMAT0019871_st | hsa-miR-4741 | 8.60 |
| 20500795 | MIMAT0004614_st | hsa-miR-193a-5p | 8.59 |
| 20500472 | MIMAT0000271_st | hsa-miR-214-3p | 8.57 |
| 20529131 | MIMAT0027442_st | hsa-miR-4433b-3p | 8.54 |
| 20525503 | MIMAT0030414_st | hsa-miR-6771-5p | 8.54 |
| 20525459 | MIMAT0027398_st | hsa-miR-6749-5p | 8.53 |
| 20515637 | MIMAT0015079_st | hsa-miR-3195 | 8.52 |
| 20520569 | MIMAT0027088_st | hsa-miR-5189-3p | 8.50 |
| 20525541 | MIMAT0027480_st | hsa-miR-6790-5p | 8.48 |
| 20500556 | MIMAT0000318_st | hsa-miR-200b-3p | 8.44 |
| 20518843 | MIMAT0018985_st | hsa-miR-3135b | 8.42 |
| 20506801 | MIMAT0005871_st | hsa-miR-1207-5p | 8.36 |
| 20500787 | MIMAT0000455_st | hsa-miR-185-5p | 8.34 |
| 20500399 | MIMAT0000231_st | hsa-miR-199a-5p | 8.33 |
| 20509227 | MIMAT0022967_st | hsa-miR-1909-3p | 8.32 |
| 20517833 | MIMAT0007883_st | hsa-miR-3620-5p | 8.32 |
| 20504295 | MIMAT0003237_st | hsa-miR-572 | 8.32 |
| 20500173 | MIMAT0000093_st | hsa-miR-93-5p | 8.30 |
| 20501287 | MIMAT0000757_st | hsa-miR-151a-3p | 8.28 |
| 20500446 | MIMAT0000257_st | hsa-miR-181b-5p | 8.26 |
| 20502446 | MIMAT0001631_st | hsa-miR-451a | 8.26 |
| 20519626 | MIMAT0027412_st | hsa-miR-4758-5p | 8.23 |
| 20525473 | MIMAT0019903_st | hsa-miR-6756-5p | 8.23 |
| 20523017 | MIMAT0023710_st | hsa-miR-6085 | 8.20 |
| 20525633 | MIMAT0027496_st | hsa-miR-6780b-5p | 8.18 |
| 20525557 | MIMAT0027572_st | hsa-miR-6798-5p | 8.18 |
| 20519405 | MIMAT0022977_st | hsa-miR-4632-5p | 8.13 |
| 20502237 | MIMAT0019041_st | hsa-miR-20b-5p | 8.10 |
| 20518901 | MIMAT0001413_st | hsa-miR-4505 | 8.10 |
| 20525549 | MIMAT0027488_st | hsa-miR-6794-5p | 8.09 |
| 20503105 | MIMAT0002177_st | hsa-miR-486-5p | 8.08 |
| 20500459 | MIMAT0000264_st | hsa-miR-203a | 8.06 |
| 20506006 | MIMAT0022938_st | hsa-miR-937-5p | 8.04 |
| 20518801 | MIMAT0018944_st | hsa-miR-4429 | 8.03 |
| 20523007 | MIMAT0023700_st | hsa-miR-6075 | 7.96 |
| 20500758 | MIMAT0000438_st | hsa-miR-152-3p | 7.94 |
| 20519615 | MIMAT0019892_st | hsa-miR-371b-5p | 7.87 |
| 20504274 | MIMAT0003218_st | hsa-miR-92b-3p | 7.87 |
| 20500462 | MIMAT0000266_st | hsa-miR-205-5p | 7.85 |
| 20500126 | MIMAT0000068_st | hsa-miR-15a-5p | 7.83 |
| 20504316 | MIMAT0003251_st | hsa-miR-548a-3p | 7.81 |
| 20518625 | MIMAT0018352_st | hsa-miR-3937 | 7.76 |
| 20506781 | MIMAT0000255_st | hsa-miR-1233-5p | 7.74 |
| 20500442 | MIMAT0022943_st | hsa-miR-34a-5p | 7.74 |
| 20518881 | MIMAT0019021_st | hsa-miR-4487 | 7.73 |
| 20500163 | MIMAT0000088_st | hsa-miR-30a-3p | 7.70 |
| 20518839 | MIMAT0018981_st | hsa-miR-4459 | 7.69 |
| 20519429 | MIMAT0019711_st | hsa-miR-4649-5p | 7.66 |
| 20525535 | MIMAT0027474_st | hsa-miR-6787-5p | 7.64 |
| 20515610 | MIMAT0015058_st | hsa-miR-3180-3p | 7.63 |
| 20519497 | MIMAT0019778_st | hsa-miR-4689 | 7.62 |
| 20504273 | MIMAT0004792_st | hsa-miR-92b-5p | 7.62 |
| 20506865 | MIMAT0015072_st | hsa-miR-1268a | 7.59 |
| 20515627 | MIMAT0005922_st | hsa-miR-320e | 7.59 |
| 20515624 | MIMAT0015070_st | hsa-miR-3188 | 7.53 |
| 20501237 | MIMAT0000728_st | hsa-miR-375 | 7.52 |
| 20506763 | MIMAT0005572_st | hsa-miR-1225-5p | 7.51 |
| 20525578 | MIMAT0027517_st | hsa-miR-6808-3p | 7.51 |
| 20503809 | MIMAT0002820_st | hsa-miR-497-5p | 7.49 |
| 20529139 | MIMAT0030422_st | hsa-miR-7847-3p | 7.49 |
| 20506779 | MIMAT0005586_st | hsa-miR-1231 | 7.46 |
| 20518904 | MIMAT0019044_st | hsa-miR-4507 | 7.44 |
| 20500158 | MIMAT0019691_st | hsa-miR-28-5p | 7.42 |
| 20501170 | MIMAT0000085_st | hsa-miR-34c-5p | 7.42 |
| 20519409 | MIMAT0000686_st | hsa-miR-4634 | 7.42 |
| 20500432 | MIMAT0000250_st | hsa-miR-139-5p | 7.34 |
| 20525519 | MIMAT0027458_st | hsa-miR-6779-5p | 7.32 |
| 20528493 | MIMAT0029782_st | hsa-miR-7641 | 7.30 |
| 20500164 | MIMAT0000089_st | hsa-miR-31-5p | 7.27 |
| 20518807 | MIMAT0019885_st | hsa-miR-4433-3p | 7.26 |
| 20519607 | MIMAT0018949_st | hsa-miR-4749-5p | 7.26 |
| 20518795 | MIMAT0018938_st | hsa-miR-548ac | 7.25 |
| 20518782 | MIMAT0018925_st | hsa-miR-1268b | 7.24 |
| 20501171 | MIMAT0004677_st | hsa-miR-34c-3p | 7.23 |
| 20500450 | MIMAT0000259_st | hsa-miR-182-5p | 7.22 |
| 20518425 | MIMAT0018178_st | hsa-miR-3180 | 7.21 |
| 20519552 | MIMAT0019833_st | hsa-miR-4720-5p | 7.18 |
| 20525565 | MIMAT0027504_st | hsa-miR-6802-5p | 7.12 |
| 20526172 | MIMAT0028111_st | hsa-miR-7107-5p | 7.10 |
| 20505787 | MIMAT0031016_st | hsa-miR-744-5p | 7.08 |
| 20529799 | MIMAT0004945_st | hsa-miR-8089 | 7.08 |
| 20529773 | MIMAT0030990_st | hsa-miR-8063 | 7.02 |
| 20500154 | MIMAT0003393_st | hsa-miR-26b-5p | 6.98 |
| 20502129 | MIMAT0000083_st | hsa-miR-425-5p | 6.98 |
| 20500421 | MIMAT0000243_st | hsa-miR-148a-3p | 6.97 |
| 20501182 | MIMAT0000692_st | hsa-miR-30e-5p | 6.96 |
| 20500159 | MIMAT0004502_st | hsa-miR-28-3p | 6.94 |
| 20501083 | MIMAT0000646_st | hsa-miR-155-5p | 6.92 |
| 20518880 | MIMAT0019020_st | hsa-miR-4486 | 6.90 |
| 20504408 | MIMAT0003322_st | hsa-miR-652-3p | 6.90 |
| 20501163 | MIMAT0021120_st | hsa-miR-200a-3p | 6.87 |
| 20520568 | MIMAT0000682_st | hsa-miR-5189-5p | 6.87 |
| 20525743 | MIMAT0027682_st | hsa-miR-6891-5p | 6.86 |
| 20517899 | MIMAT0018068_st | hsa-miR-3648 | 6.85 |
| 20518818 | MIMAT0018961_st | hsa-miR-4443 | 6.85 |
| 20502124 | MIMAT0001340_st | hsa-miR-423-3p | 6.83 |
| 20519457 | MIMAT0019887_st | hsa-miR-4665-5p | 6.83 |
| 20519609 | MIMAT0019739_st | hsa-miR-4750-5p | 6.83 |
| 20525525 | MIMAT0027464_st | hsa-miR-6782-5p | 6.77 |
| 20504342 | MIMAT0003270_st | hsa-miR-602 | 6.76 |
| 20525574 | MIMAT0027513_st | hsa-miR-6806-3p | 6.69 |
| 20500748 | MIMAT0000681_st | hsa-miR-141-3p | 6.67 |
| 20501160 | MIMAT0000732_st | hsa-miR-29c-3p | 6.67 |
| 20501243 | MIMAT0000432_st | hsa-miR-378a-3p | 6.67 |
| 20515623 | MIMAT0015069_st | hsa-miR-3187-3p | 6.63 |
| 20525585 | MIMAT0027524_st | hsa-miR-6812-5p | 6.62 |
| 20502123 | MIMAT0004748_st | hsa-miR-423-5p | 6.61 |
| 20525523 | MIMAT0027462_st | hsa-miR-6781-5p | 6.59 |
| 20526178 | MIMAT0028117_st | hsa-miR-7110-5p | 6.57 |
| 20517680 | MIMAT0016852_st | hsa-miR-4298 | 6.56 |
| 20526861 | MIMAT0028211_st | hsa-miR-7150 | 6.56 |
| 20525383 | MIMAT0025844_st | hsa-miR-6716-5p | 6.55 |
| 20525599 | MIMAT0027538_st | hsa-miR-6819-5p | 6.54 |
| 20525719 | MIMAT0027658_st | hsa-miR-6879-5p | 6.52 |
| 20506901 | MIMAT0005951_st | hsa-miR-1307-3p | 6.50 |
| 20501168 | MIMAT0000685_st | hsa-miR-34b-5p | 6.45 |
| 20519524 | MIMAT0019806_st | hsa-miR-4706 | 6.45 |
| 20503808 | MIMAT0002819_st | hsa-miR-193b-3p | 6.42 |
| 20525609 | MIMAT0027548_st | hsa-miR-6824-5p | 6.42 |
| 20525681 | MIMAT0027620_st | hsa-miR-6769b-5p | 6.39 |
| 20529137 | MIMAT0030420_st | hsa-miR-7845-5p | 6.37 |
| 20525395 | MIMAT0025855_st | hsa-miR-6723-5p | 6.31 |
| 20515585 | MIMAT0015036_st | hsa-miR-3162-5p | 6.29 |
| 20519498 | MIMAT0019779_st | hsa-miR-4690-5p | 6.27 |
| 20500132 | MIMAT0000072_st | hsa-miR-18a-5p | 6.23 |
| 20500120 | MIMAT0004484_st | hsa-let-7d-3p | 6.21 |
| 20506872 | MIMAT0000460_st | hsa-miR-1275 | 6.20 |
| 20500797 | MIMAT0005929_st | hsa-miR-194-5p | 6.20 |
| 20520577 | MIMAT0021128_st | hsa-miR-5196-5p | 6.20 |
| 20506839 | MIMAT0022721_st | hsa-miR-1247-3p | 6.19 |
| 20501169 | MIMAT0004676_st | hsa-miR-34b-3p | 6.16 |
| 20525487 | MIMAT0027426_st | hsa-miR-6763-5p | 6.12 |
| 20506009 | MIMAT0004982_st | hsa-miR-939-5p | 6.12 |
| 20525384 | MIMAT0025845_st | hsa-miR-6716-3p | 6.09 |
| 20525644 | MIMAT0027583_st | hsa-miR-6840-3p | 6.09 |
| 20518842 | MIMAT0018984_st | hsa-miR-378h | 6.08 |
| 20517816 | MIMAT0017986_st | hsa-miR-3609 | 6.05 |
| 20500395 | MIMAT0000227_st | hsa-miR-197-3p | 6.04 |
| 20505790 | MIMAT0004948_st | hsa-miR-885-3p | 6.04 |
| 20501279 | MIMAT0004694_st | hsa-miR-342-5p | 6.03 |
| 20518826 | MIMAT0018968_st | hsa-miR-4449 | 6.03 |
