# Supplementary material for: A modified protocol for successful miRNA profiling in human precision-cut lung slices (PCLS)
Source: BMC Res Notes. 2021 Jul 2;14:255. doi: 10.1186/s13104-021-05674-w (PMC8252208; doi:10.1186/s13104-021-05674-w)
Supplement: Supplementary file 4 — Additional file 4: Table S3. A selected list of some miRNAs detected in human PCLS with already described function in pulmonary diseases. [file 13104_2021_5674_MOESM4_ESM.docx]

**Niehof et al. A modified protocol for successful miRNA profiling in human precision-cut lung slices (PCLS)**

**Additional file 4. Table S3.**

**Table S3**. A selected list of miRNAs detected in human PCLS with already described function in pulmonary diseases

| **Array ID** | **Transcript ID** | **Lung development  & function** | **IPF** | **COPD** | **Asthma** | **Lung cancer** |
| --- | --- | --- | --- | --- | --- | --- |
| 20500112 | hsa-let-7a-5p |  | [1, 2] |  | [2, 3] | [2] |
| 20500115 | hsa-let-7b-5p |  | [1, 2, 4] |  | [2, 5] | [2] |
| 20500117 | hsa-let-7c-5p |  | [1] | [3, 5] |  |  |
| 20500119 | hsa-let-7d-5p | [5, 6] | [1, 7–10] |  | [6] | [6] |
| 20500123 | hsa-let-7f-5p |  |  |  | [3] |  |
| 20500713 | hsa-let-7g-5p |  | [1] |  |  |  |
| 20500183 | hsa-miR-100-5p |  | [1] |  |  | [5] |
| 20500191 | hsa-miR-103a-3p |  | [1] |  |  |  |
| 20500194 | hsa-miR-106a-5p | [5] | [1] |  | [2–4, 6, 7] | [6] |
| 20500438 | hsa-miR-10a-5p |  |  |  |  | [11] |
| 20500765 | hsa-miR-125a-5p |  | [1] | [5] |  |  |
| 20500730 | hsa-miR-125b-5p |  | [1, 7] | [5, 6] |  |  |
| 20500769 | hsa-miR-126-3p |  | [1, 4] |  | [2–7] | [5] |
| 20500746 | hsa-miR-140-3p |  | [1] |  |  |  |
| 20500755 | hsa-miR-145-5p | [6] | [2, 8–10] | [3, 4] | [2–7] |  |
| 20500778 | hsa-miR-146a-5p | [6] | [1] |  | [4, 6, 7] |  |
| 20503793 | hsa-miR-146b-5p | [6] | [1] |  | [3, 6, 7] | [2] |
| 20500781 | hsa-miR-149-3p |  |  | [4] |  |  |
| 20501083 | hsa-miR-155-5p | [6] | [1, 7] |  | [3, 4, 7] | [4–6] |
| 20500126 | hsa-miR-15a-5p |  |  |  |  | [6] |
| 20500718 | hsa-miR-15b-5p | [6] |  | [4] |  | [6] |
| 20500128 | hsa-miR-16-5p | [6] |  |  | [5] | [2, 4, 6] |
| 20500130 | hsa-miR-17-5p | [5] | [1, 7, 8, 10] | [3] | [6] | [4, 6] |
| 20500444 | hsa-miR-181a-5p |  | [1] | [3] | [3, 4, 6] |  |
| 20500450 | hsa-miR-182-5p |  |  |  |  | [11] |
| 20500761 | hsa-miR-191-5p |  |  | [5] |  | [11, 12] |
| 20500795 | hsa-miR-193a-5p |  |  |  |  | [4] |
| 20500798 | hsa-miR-195-5p | [6, 7] |  |  |  |  |
| 20500400 | hsa-miR-199a-3p |  | [2, 8, 10] | [3, 4] |  |  |
| 20500137 | hsa-miR-19b-3p |  | [1] |  |  | [13] |
| 20500556 | hsa-miR-200b-3p |  | [8, 10] |  |  | [5, 6] |
| 20501036 | hsa-miR-200c-3p | [6, 7] | [2, 8, 10] |  |  | [5] |
| 20500462 | hsa-miR-205-5p |  |  |  |  | [5, 6] |
| 20500139 | hsa-miR-20a-5p | [5] | [1] |  |  |  |
| 20502237 | hsa-miR-20b-5p |  |  |  | [6, 7] |  |
| 20500141 | hsa-miR-21-5p |  | [2, 4] | [3] | [2–4, 6, 7] | [2, 4, 6] |
| 20500484 | hsa-miR-221-3p |  | [1] |  | [2–7] | [2, 5, 6] |
| 20500486 | hsa-miR-222-3p |  | [1] |  | [4–7] | [2, 5, 6] |
| 20500144 | hsa-miR-22-3p |  | [1] |  |  |  |
| 20500146 | hsa-miR-23a-3p |  |  |  |  | [14] |
| 20500148 | hsa-miR-24-3p |  |  |  | [3] |  |
| 20500151 | hsa-miR-25-3p |  |  |  | [2] |  |
| 20500152 | hsa-miR-26a-5p | [5, 6] | [1, 8–10] |  | [2] |  |
| 20500157 | hsa-miR-27a-3p |  | [10] |  | [3] | [2] |
| 20500723 | hsa-miR-27b-3p |  | [1] |  | [3] |  |
| 20500161 | hsa-miR-29a-3p | [2, 5, 6] | [1, 7–10] |  | [3] | [6] |
| 20501160 | hsa-miR-29c-3p | [2] | [1, 2, 7–10] |  |  |  |
| 20500162 | hsa-miR-30a-5p |  | [1] |  |  |  |
| 20500724 | hsa-miR-30b-5p |  | [1] |  |  |  |
| 20500422 | hsa-miR-30c-5p |  |  | [3, 5] | [5] | [5] |
| 20501277 | hsa-miR-328-5p |  |  |  | [3] |  |
| 20501280 | hsa-miR-342-3p |  | [1] |  |  |  |
| 20500442 | hsa-miR-34a-5p |  | [10] |  |  | [2, 6] |
| 20501237 | hsa-miR-375 |  | [10] |  |  |  |
| 20502124 | hsa-miR-423-3p |  | [15] |  |  |  |
| 20502123 | hsa-miR-423-5p |  | [15] |  |  |  |
| 20519525 | hsa-miR-4707-5p |  |  |  | [3] |  |
| 20504316 | hsa-miR-548a-3p |  |  |  |  | [6] |
| 20504391 | hsa-miR-638 |  |  | [3] |  |  |
| 20500171 | hsa-miR-92a-3p |  | [1] |  |  |  |
| 20506006 | hsa-miR-937-5p |  |  | [6, 7] |  |  |
| 20500181 | hsa-miR-99a-5p |  |  | [5] |  |  |
| 20501176 | hsa-miR-99b-5p |  |  | [5] |  |  |

References

1. Bagnato G, Roberts WN, Roman J, Gangemi S. A systematic review of overlapping microRNA patterns in systemic sclerosis and idiopathic pulmonary fibrosis. Eur Respir Rev 2017. doi:10.1183/16000617.0125-2016.

2. Booton R, Lindsay MA. Emerging role of MicroRNAs and long noncoding RNAs in respiratory disease. Chest. 2014;146:193–204. doi:10.1378/chest.13-2736.

3. Szymczak I, Wieczfinska J, Pawliczak R. Molecular Background of miRNA Role in Asthma and COPD: An Updated Insight. Biomed Res Int. 2016;2016:7802521. doi:10.1155/2016/7802521.

4. Dutta RK, Chinnapaiyan S, Unwalla H. Aberrant MicroRNAomics in Pulmonary Complications: Implications in Lung Health and Diseases. Mol Ther Nucleic Acids. 2019;18:413–31. doi:10.1016/j.omtn.2019.09.007.

5. Sittka A, Schmeck B. MicroRNAs in the lung. Adv Exp Med Biol. 2013;774:121–34. doi:10.1007/978-94-007-5590-1_7.

6. Alipoor SD, Adcock IM, Garssen J, Mortaz E, Varahram M, Mirsaeidi M, Velayati A. The roles of miRNAs as potential biomarkers in lung diseases. Eur J Pharmacol. 2016;791:395–404. doi:10.1016/j.ejphar.2016.09.015.

7. Sessa R, Hata A. Role of microRNAs in lung development and pulmonary diseases. Pulm Circ. 2013;3:315–28. doi:10.4103/2045-8932.114758.

8. Pandit KV, Milosevic J. MicroRNA regulatory networks in idiopathic pulmonary fibrosis. Biochem Cell Biol. 2015;93:129–37. doi:10.1139/bcb-2014-0101.

9. Cho SJ, Lee M, Stout-Delgado HW, Moon J-S. DROSHA-Dependent miRNA and AIM2 Inflammasome Activation in Idiopathic Pulmonary Fibrosis. Int J Mol Sci 2020. doi:10.3390/ijms21051668.

10. Wang Y, Xiao H, Zhao F, Li H, Gao R, Yan B, et al. Decrypting the crosstalk of noncoding RNAs in the progression of IPF. Mol Biol Rep. 2020;47:3169–79. doi:10.1007/s11033-020-05368-9.

11. Raponi M, Dossey L, Jatkoe T, Wu X, Chen G, Fan H, Beer DG. MicroRNA classifiers for predicting prognosis of squamous cell lung cancer. Cancer Res. 2009;69:5776–83. doi:10.1158/0008-5472.CAN-09-0587.

12. Manikandan J, Aarthi JJ, Kumar SD, Pushparaj PN. Oncomirs: the potential role of non-coding microRNAs in understanding cancer. Bioinformation. 2008;2:330–4. doi:10.6026/97320630002330.

13. Hwang H-W, Mendell JT. MicroRNAs in cell proliferation, cell death, and tumorigenesis. Br J Cancer. 2006;94:776–80. doi:10.1038/sj.bjc.6603023.

14. Slaby O, Svoboda M, Michalek J, Vyzula R. MicroRNAs in colorectal cancer: translation of molecular biology into clinical application. Mol Cancer. 2009;8:102. doi:10.1186/1476-4598-8-102.

15. Oak SR, Murray L, Herath A, Sleeman M, Anderson I, Joshi AD, et al. A micro RNA processing defect in rapidly progressing idiopathic pulmonary fibrosis. PLoS One. 2011;6:e21253. doi:10.1371/journal.pone.0021253.
