# Supplementary material for: A modified protocol for successful miRNA profiling in human precision-cut lung slices (PCLS)
Source: BMC Res Notes. 2021 Jul 2;14:255. doi: 10.1186/s13104-021-05674-w (PMC8252208; doi:10.1186/s13104-021-05674-w)
Supplement: Supplementary file 5 — Additional file 5: Table S4. List of filtered 75 differentially-expressed miRNAs in comparison T1-HD versus control used as basis during hierarchical clustering analysis. [file 13104_2021_5674_MOESM5_ESM.docx]

**Niehof et al. A modified protocol for successful miRNA profiling in human precision-cut lung slices (PCLS)**

**Additional file 5. Table S4.**

**Table S4.** List of filtered 75 differentially-expressed miRNAs in comparison T1-HD versus control used as basis during hierarchical clustering analysis

| **Array ID** | **T1-HD**  **Avg (log2)** | **CTRL**  **Avg (log2)** | **Fold Change** | **P-val** | **Transcript ID  (Array Design)** |
| --- | --- | --- | --- | --- | --- |
| 20517916 | 5.86 | 4.75 | 2.17 | 0.0491 | hsa-miR-3663-3p |
| 20519410 | 4.81 | 2.9 | 3.77 | 0.0437 | hsa-miR-4635 |
| 20500777 | 3.63 | 2.47 | 2.23 | 0.0422 | hsa-miR-138-1-3p |
| 20525438 | 4.32 | 2.68 | 3.12 | 0.0402 | hsa-miR-6738-5p |
| 20500143 | 5.32 | 3.57 | 3.37 | 0.0385 | hsa-miR-22-5p |
| 20517821 | 4.46 | 1.81 | 6.27 | 0.0382 | hsa-miR-3613-3p |
| 20535133 | 4.72 | 3.59 | 2.18 | 0.0379 | hsa-mir-484 |
| 20500796 | 4.03 | 2.26 | 3.42 | 0.0378 | hsa-miR-193a-3p |
| 20500743 | 5.13 | 3.49 | 3.12 | 0.0368 | hsa-miR-138-5p |
| 20500448 | 6.66 | 4.71 | 3.87 | 0.0357 | hsa-miR-181c-5p |
| 20501229 | 3.74 | 2.7 | 2.06 | 0.0355 | hsa-miR-371a-5p |
| 20500471 | 4.2 | 2.75 | 2.74 | 0.0328 | hsa-miR-214-5p |
| 20510799 | 1.45 | 3.85 | -5.28 | 0.0326 | hsa-miR-1972 |
| 20519702 | 4.33 | 2.6 | 3.31 | 0.0313 | hsa-miR-4800-3p |
| 20532675 | 7.09 | 8.28 | -2.28 | 0.0305 | ACA44 |
| 20534056 | 7.09 | 8.28 | -2.28 | 0.0305 | ENSG00000252840 |
| 20500149 | 5.81 | 4.76 | 2.06 | 0.0302 | hsa-miR-24-2-5p |
| 20504584 | 4 | 2.59 | 2.66 | 0.0295 | hsa-miR-378d |
| 20501201 | 4.68 | 3.25 | 2.68 | 0.0266 | hsa-miR-362-5p |
| 20502451 | 5.04 | 3.34 | 3.24 | 0.0250 | hsa-miR-452-5p |
| 20502130 | 3.85 | 2.74 | 2.16 | 0.0250 | hsa-miR-425-3p |
| 20521783 | 3.22 | 1.74 | 2.79 | 0.0234 | hsa-miR-5571-5p |
| 20517745 | 3.74 | 2.26 | 2.79 | 0.0227 | hsa-miR-4286 |
| 20517675 | 6.31 | 5.12 | 2.28 | 0.0220 | hsa-miR-378c |
| 20534320 | 2.76 | 1.51 | 2.38 | 0.0218 | HBII-85-26 |
| 20538308 | 3.67 | 5.09 | -2.67 | 0.0216 | ENSG00000238498 |
| 20532695 | 2.76 | 3.77 | -2.01 | 0.0216 | ACA57 |
| 20500483 | 4.88 | 2.65 | 4.69 | 0.0214 | hsa-miR-221-5p |
| 20500131 | 4.64 | 2.78 | 3.65 | 0.0193 | hsa-miR-17-3p |
| 20504290 | 3.77 | 2.18 | 3.02 | 0.0191 | hsa-miR-551b-3p |
| 20500156 | 4.11 | 2.35 | 3.38 | 0.0180 | hsa-miR-27a-5p |
| 20537519 | 4.22 | 5.54 | -2.49 | 0.0177 | hsa-mir-6776 |
| 20525711 | 3.65 | 2.51 | 2.21 | 0.0173 | hsa-miR-6875-5p |
| 20501159 | 3.72 | 2.5 | 2.33 | 0.0163 | hsa-miR-29c-5p |
| 20519549 | 2.47 | 3.79 | -2.48 | 0.0154 | hsa-miR-4717-3p |
| 20518794 | 4.44 | 2.54 | 3.73 | 0.0149 | hsa-miR-378g |
| 20500489 | 5.54 | 3.67 | 3.65 | 0.0147 | hsa-miR-224-5p |
| 20520351 | 9.69 | 11.03 | -2.52 | 0.0146 | hsa-miR-1273g-3p |
| 20532694 | 2.69 | 3.87 | -2.27 | 0.0143 | ACA57 |
| 20515618 | 4.44 | 2.65 | 3.45 | 0.0130 | hsa-miR-3065-5p |
| 20501309 | 2.45 | 1.37 | 2.12 | 0.0112 | hsa-miR-133b |
| 20503102 | 4.48 | 3.39 | 2.13 | 0.0112 | hsa-miR-484 |
| 20500457 | 4.69 | 2.07 | 6.16 | 0.0103 | hsa-miR-199b-5p |
| 20532596 | 4.06 | 5.32 | -2.4 | 0.0092 | 14qII-24 |
| 20506857 | 2.01 | 4.97 | -7.77 | 0.0092 | hsa-miR-1263 |
| 20502122 | 5.9 | 4.52 | 2.61 | 0.0092 | hsa-miR-422a |
| 20525562 | 2.21 | 4.03 | -3.52 | 0.0086 | hsa-miR-6800-3p |
| 20503786 | 5.01 | 2.78 | 4.69 | 0.0082 | hsa-miR-489-3p |
| 20504186 | 4.24 | 1.7 | 5.81 | 0.0082 | hsa-miR-455-5p |
| 20500174 | 3.38 | 1.96 | 2.68 | 0.0071 | hsa-miR-93-3p |
| 20500443 | 4.34 | 1.93 | 5.31 | 0.0060 | hsa-miR-34a-3p |
| 20500751 | 3.82 | 2.07 | 3.35 | 0.0055 | hsa-miR-143-5p |
| 20532564 | 2.57 | 3.59 | -2.04 | 0.0046 | 14qI-1 |
| 20533759 | 1.71 | 2.96 | -2.38 | 0.0036 | ENSG00000239155 |
| 20519463 | 4.79 | 1.36 | 10.76 | 0.0032 | hsa-miR-4668-5p |
| 20525675 | 2.42 | 1.23 | 2.28 | 0.0028 | hsa-miR-6857-5p |
| 20518919 | 5.71 | 2.97 | 6.66 | 0.0026 | hsa-miR-4521 |
| 20504316 | 5.83 | 7.81 | -3.94 | 0.0024 | hsa-miR-548a-3p |
| 20529566 | 3.82 | 2.66 | 2.23 | 0.0023 | hsa-miR-7975 |
| 20504187 | 8.62 | 9.78 | -2.23 | 0.0022 | hsa-miR-455-3p |
| 20501175 | 3.76 | 1.71 | 4.14 | 0.0020 | hsa-miR-301a-3p |
| 20500739 | 2.56 | 1.31 | 2.38 | 0.0018 | hsa-miR-133a-3p |
| 20501242 | 2.83 | 1.25 | 2.99 | 0.0016 | hsa-miR-378a-5p |
| 20533693 | 5.7 | 7.61 | -3.76 | 0.0002 | ENSG00000239055 |
| 20533451 | 0.75 | 1.94 | -2.28 | 0.0001 | ENSG00000238686 |
| 20533712 | 5.99 | 7.71 | -3.3 | 4.21E-05 | ENSG00000239080 |
| 20533450 | 1.02 | 2.43 | -2.66 | 4.04E-05 | ENSG00000238686 |
| 20533260 | 7.32 | 9.58 | -4.79 | 2.51E-05 | ENSG00000238388 |
| 20533259 | 7.29 | 9.68 | -5.23 | 1.69E-05 | ENSG00000238388 |
| 20533711 | 5.91 | 7.85 | -3.85 | 1.63E-05 | ENSG00000239080 |
| 20533275 | 6.23 | 8.07 | -3.59 | 1.38E-05 | ENSG00000238414 |
| 20533758 | 6.08 | 7.91 | -3.55 | 1.16E-05 | ENSG00000239154 |
| 20533836 | 3.97 | 5.43 | -2.76 | 1.01E-05 | ENSG00000251940 |
| 20534201 | 3.97 | 5.43 | -2.76 | 1.01E-05 | ENSG00000268890 |
| 20533757 | 7.19 | 9.43 | -4.71 | 9.07E-06 | ENSG00000239154 |
